# A back-translational study of descending interactions with the induction of hyperalgesia by high-frequency electrical stimulation in rats and humans

**DOI:** 10.1097/j.pain.0000000000003166

**Published:** 2024-01-09

**Authors:** Ryan Patel, Joseph L. Taylor, Anthony H. Dickenson, Stephen B. McMahon, Kirsty Bannister

**Affiliations:** a King's College London, Wolfson Centre, Guy's Campus, London, United Kingdom; b Department of Neuroscience, Physiology & Pharmacology, University College London, London, United Kingdom

**Keywords:** Conditioned pain modulation, Quantitative sensory testing, Secondary hyperalgesia, High-frequency electrocutaneous stimulation, In vivo electrophysiology, Dorsal horn, Wide dynamic range, Diffuse noxious inhibitory controls

## Abstract

In humans and animals, high-frequency electrocutaneous stimulation (HFS) induces an “early long-term potentiation-like” sensitisation, where synaptic plasticity is underpinned by an ill-defined interaction between peripheral input and central modulatory processes. The relative contributions of these processes to the initial pain or nociceptive response likely differ from those that underpin development of the heightened response. To investigate the impact of HFS-induced hyperalgesia on pain and nociception in perception and neural terms, respectively, and to explore the impact of descending inhibitory pathway activation on the development of HFS-induced hyperalgesia, we performed parallel studies utilising identical stimuli to apply HFS concurrent to (1) a conditioned pain modulation paradigm during psychophysical testing in healthy humans or (2) a diffuse noxious inhibitory controls paradigm during in vivo electrophysiological recording of spinal neurones in healthy anaesthetised rats. High-frequency electrocutaneous stimulation alone induced enhanced perceptual responses to pinprick stimuli in cutaneous areas secondary to the area of electrical stimulation in humans and increased the excitability of spinal neurones which exhibited stimulus intensity–dependent coded responses to pinprick stimulation in a manner that tracked with human psychophysics, supporting their translational validity. Application of a distant noxious conditioning stimulus during HFS did not alter perceived primary or secondary hyperalgesia in humans or the development of primary or secondary neuronal hyperexcitability in rats compared with HFS alone, suggesting that, upon HFS-response initiation in a healthy nervous system, excitatory signalling escapes inhibitory control. Therefore, in this model, dampening facilitatory mechanisms rather than augmenting top-down inhibitions could prevent pain development.

## Abstract 2

High-frequency electrocutaneous stimulation induces perceptual and neuronal correlates of hyperalgesia, and concurrently applying a distant conditioning stimulus does not affect the development of sensitisation.

## 1. Introduction

Human surrogate models of pain have the potential to bridge the gap between pre-clinical and clinical research. Typically surrogate models are used to mimic the positive sensory phenomena associated with chronic pain states.^31^ However, crucial to successful translation is the predictive value of animal models when assessing underlying mechanisms. Tetanic stimulation of C-fibres has long been known to elicit homosynaptic and heterosynaptic long-term potentiation (LTP) in lamina I of the dorsal horn,^22,32^ which is proposed as a prospective mechanism underlying activity-dependent spinal plasticity in chronic pain states.^14^ In addition to relaying increased nociceptive activity to higher centres, NK1+ lamina I neurones are the origin of a spino-bulbo-spinal loop controlling deep dorsal horn neuronal excitability after induction of LTP.^34,38^ The activity of these deep dorsal horn neurones whose firing increases substantially on application of a noxious stimulus may also govern nociceptive amplification.^34,39^ “Early LTP-like” phenomena have been described in humans after high-frequency stimulation (HFS), and the resultant primary and secondary hyperalgesia are hypothesised to reflect perceptual correlates of homosynaptic and heterosynaptic facilitation.^10,17^

From a functional perspective excitatory and inhibitory mechanisms likely exist in a counterbalanced manner to fine tune spinal transmission. Although spinal neuronal sensitisation can be heavily dependent on the nature of peripheral excitatory drive and subsequent synaptic plasticity, the overall resulting hyperexcitability will be determined by the relative strengths of local (propriospinal) and descending excitatory and inhibitory mechanisms. Reversible spinal block of descending tracts increases spinal neuronal excitability posttetanic stimulation suggesting that descending inhibitions, in the context of inducing LTP, may act to restrict spatially and temporally the spread of sensitisation.^8^ Because the individual physiological response to pain arises from various combinations of excitatory and inhibitory mechanisms at multiple levels throughout the sensory neuroaxis, there are contrasting strategies for pain management. Central to the development of rational pain treatment strategies is not only the assessment of pain mechanisms but their interaction. One way in which this can be investigated is via the application of protocols that combine peripheral nervous system and modulatory pathway stimulation.

In this study, we began by performing rat neurophysiological recordings of wide-dynamic range (WDR) neuronal activity alongside human psychophysics using parallel techniques to induce hyperalgesia with HFS to verify any concordance between perceptual and neuronal responses in humans and rats, respectively. Then, we examined using identical test paradigms whether applying a conditioning stimulus to evoke activity in a descending inhibitory control pathway concurrent to HFS affected (1) the development of secondary hyperalgesia in healthy pain-free subjects (representing a conditioned pain modulation [CPM] paradigm) or (2) neurophysiological responses of WDR neurones in healthy anaesthetised rats (representing a diffuse noxious inhibitory controls [DNIC] paradigm). Because of the convergence of peripheral and descending mechanisms on WDR neurones, they are aptly placed in the sensory pathway to provide a readout of neuronal substrates of peripheral and central sensitisation in a manner that relates to sensory testing measures to the same stimuli.^26,35^

## 2. Methods

### 2.1. Subjects for the quantitative sensory testing study

Thirty-seven healthy subjects were included in the study (8 men: 25.8 ± 5.03 years [range 20-41 years]; 29 women: 25.4 ± 2.81 years [range 18-50 years]). Information about the study was disseminated both via the King's College London fortnightly research volunteers email circular and within the department. Inclusion criteria specified that subjects should be between 18 and 70 years old, have a strong grasp of English, and be free of pain and medication (except contraception) on the day of testing. Exclusion criteria included acute or chronic pain conditions, dermatological issues at the site of testing, pregnancy, neurological disorders, and musculoskeletal or inflammatory conditions. Subjects were requested not to consume alcohol in the 24-hour period before testing and to avoid excessively strenuous exercise of the legs. All subjects provided informed consent before testing that took place within the Wolfson Centre. The study was approved by King's College London Research Ethics Committee (Reference RESCM-21/22-22208) and performed according to the Helsinki Declaration.^45^

### 2.2. Protocol for quantitative sensory testing

**Figure 1.**
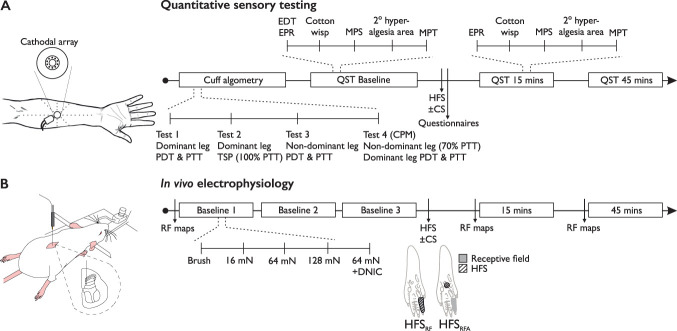
Schematics of experimental sessions. (A) Timeline depicting procedure for HFS (control) and HFS (+CS) quantitative sensory testing sessions in humans. (B) Timeline depicting experimental protocol for rat in vivo electrophysiology. CPM, conditioned pain modulation; CS, conditioning stimulus; DNIC, diffuse noxious inhibitory controls; EDT, electrical detection threshold; EPR, electrical pain rating; HFS, high-frequency stimulation; MPS, mechanical pain sensitivity; MPT, mechanical pain threshold; PDT, pain detection threshold; PTT, pressure tolerance threshold; QST, quantitative sensory testing; RF, receptive field; RFA, receptive field adjacent; TSP, temporal summation of pain.

Subjects attended 2 sessions that lasted approximately 2 hours. These sessions were set at least 2 days apart to avoid any confounding effects of long-lasting sensitisation. During the HFS (control) session, subjects received HFS in isolation, whereas in the HFS (+CS) session, subjects received HFS at the same time as a conditioning stimulus (CS) in the form of tonic pressure to the contralateral calf (Fig. 1A). Session order was randomised.

#### 2.2.1. Conditioned pain modulation and temporal summation of pain

Cuff pressure pain sensitivity and CPM were determined using a computer-controlled cuff algometry system (Nocitech CPAR, Aalborg University, Denmark) as previously described.^5^ In brief, subjects experienced a slow increasing pressure ramp (1 kPa/second up to a maximum of 100 kPa) applied to the calf and were asked to rate pain intensity continuously on a visual analogue scale (VAS) using an electronic VAS device with a sliding bar which digitised to a 0 to 10 scale. The bar was anchored visually on the controller with “min” and “max” and verbally by the experimenters as “no pain at all” and “the worst pain imaginable.” Pain detection threshold (PDT) was taken as the pressure (kPa) when a VAS score of 1 of 10 was reached, and pain tolerance threshold (PTT) was taken when subjects self-terminated the test indicating when they “cannot tolerate any more pressure.” For those who reached the maximum of 100 kPa, this was taken as their PTT. Firstly, the pressure ramp was applied to the dominant leg to determine PDT and PTT, followed by assessment of temporal summation of pain (TSP) at a frequency of 1 second on/1 second off (10 stimuli applied at 100% PTT). The pressure ramp was then applied to the nondominant leg to determine PDT and PTT. To assess CPM, an increasing pressure ramp was applied to the dominant leg and PDT and PTT were determined, whilst tonic pressure was applied to the nondominant leg (70% PTT). The conditioning pressure applied during HFS was taken as 70% of the PTT on the leg contralateral to the subject's dominant arm, where the electrical stimulation was applied, and was also applied to this leg during the electrical stimulation.

As per the recommended guidelines,^48^ the CPM effect was calculated as the baseline PDT minus the conditioned PDT measurements. This ensured that the presence of an inhibitory CPM effect was indicated by a negative value, and a facilitatory CPM effect was indicated by a positive value. The ramps were applied twice at the beginning of each session and the difference was calculated for each session, and then values from each of the 2 sessions were averaged to calculate the mean CPM effect. Classifying subjects by their CPM response was performed using the standard error of measurement^6,16^ calculated as follows: SEM = standard deviation of baseline PDT × √(1 − intraclass correlation coefficient (ICC (A,1), 2-way mixed effects) of baseline PDT). Subjects with a mean CPM response greater than the SEM were classified as CPM responders and those with a response less than the SEM as CPM nonresponders. The wind-up ratio (WUR) was calculated as the sum of pain intensity ratings evoked by all 10 stimuli divided by the theoretical nonpotentiated response (10 × the first stimulus pain intensity rating); values were averaged across the 2 sessions.

#### 2.2.2. High-frequency electrocutaneous stimulation

High-frequency electrocutaneous stimulation and psychophysical testing was performed as previously described.^17^ Subjects received electrical stimuli via an EPS-P10 electrode (cathode: 10 pins, 0.25 mm diameter, anode: 24 × 22 mm^2^; MRC Systems GmbH, Germany), which was attached to the volar part of the subject's dominant forearm (Fig. 1A). Electrical pulses were delivered transcutaneously via a DS7A High Voltage Constant Current Stimulator (Digitimer Ltd, Welwyn Garden City, United Kingdom), whereas trains of stimulation were generated using the DG2A Train Delay Generator (Digitimer Ltd). Initially after attachment of the electrode, a single 2-millisecond pulse at 1 mA was administered as a familiarisation to the subject with the electrical stimuli and to ensure correct placement of the electrode. Subjects then had their electrical detection threshold (EDT) measured using the method of limits. Individual pulses were applied, and stimulus intensity (mA) was gradually decreased until subjects no longer perceived it. The intensity was then increased until the pulses were detected again, and this was repeated 5 times. The initial value was set at 1 mA to ensure the intensity always began above detection threshold. Nondetection and detection intensities were measured to 0.01 mA resolution, and the geometric mean of the 10 recorded intensities was taken as the EDT as previously described.^33^ The intensity of the stimulation was then increased to 15 times the EDT and was used assess electrical pain ratings (EPR) for the remainder of the session. Electrical detection threshold was measured at the beginning of both sessions, but the intensity used for all electrical stimuli was determined using the EDT from the first session.

Sensitivity to mechanical stimuli was assessed with a shortened version of the DFNS mechanical pain sensitivity (MPS) protocol.^33^ This utilised only one of the original 3 non-noxious stimuli (cotton wisp) and 4 of the pinprick stimuli (32, 64, 128, and 256 mN; MRC Systems GmbH), together with single 2-millisecond electrical pulses applied at 15× EDT. Each stimulus was applied 5 times within the circular area 1 cm from the edge of the electrode in a counterbalanced pseudo-random order. Subjects were asked to rate the painfulness of each stimulus on a numerical rating scale (NRS) from 0 (no pain at all) to 100 (the worst pain imaginable). Pain ratings for each individual stimulus were averaged both individually and as a whole with the geometric mean of all the pinprick pain ratings being taken as the subjects' MPS rating. The full DFNS mechanical pain threshold (MPT) test was also performed. To sample all around the electrode, pinprick stimuli were applied in a clockwise direction around electrode in the same area as the MPS test. The reliability of baseline measures between sessions is shown in Figure S1 (available as supplemental digital content at http://links.lww.com/PAIN/B983).

To elicit secondary mechanical hyperalgesia, 5 trains of electrical stimulation (100 Hz, 2 ms pulse width, 1 second duration) were then delivered 10 seconds apart at 15× EDT. Immediately after the HFS stimulation was applied, subjects completed a measure of both state and trait anxiety^37^ and the SF-36 health questionnaire.^41^ Sensory testing was then repeated at 15 minutes and 45 minutes post-HFS. To measure the spread of hyperalgesia, a 10-g von Frey filament (Touch Test; North Coast Medical, Morgan Hill, CA) was applied at 1-cm intervals along 8 orthogonal directions around the electrode (Fig. 1A). Subjects were asked to make the same judgement as in the MPT test and indicate whether the stimulus had a “sharp, stinging or pricking sensation” or a “normal touch sensation.” Stimuli were applied from outside to in, starting at a maximum of 8 cm. The spread of hyperalgesia was taken as the number of consecutive “sharp” ratings from the centre (eg, final 3 stimulations rated as “sharp” with a “blunt” rating at 4 cm was taken as 3 cm in that direction). The distances from the centre were modelled as an irregular polygon using MATLAB (MathWorks, Natick, MA), the area of which was taken as the area of secondary hyperalgesia.

### 2.3. Animals

Adult male Lister Hooded rats (34 in total; 250-300 g) were used for electrophysiological experiments (Charles River, United Kingdom). Animals were group housed (maximum of 5) on a conventional 12:12-hour light–dark cycle; food and water were available ad libitum. Temperature (20-22°C) and humidity (55%-65%) of holding rooms were closely regulated. Experimental design/analysis was conducted according to ARRIVE guidelines. All procedures described here were approved by an internal ethics panel and the UK Home Office (licence PABEF3413) under the Animals (Scientific Procedures) Act 1986.

### 2.4. In vivo electrophysiology

Anaesthesia was initially induced with 3.5% vol/vol isoflurane delivered in 3:2 ratio of nitrous oxide and oxygen. Once areflexic, a tracheotomy was performed and rats were subsequently maintained on 1.5% vol/vol isoflurane for the remainder of the experiment (approximately 3-4 hours; core body temperature was maintained throughout with the use of a homeothermic blanket). Rats were secured in a stereotaxic frame and a laminectomy was performed to expose the L4-L6 segments of the spinal cord; 2 spinal clamps were applied to stabilise the spinal column. Extracellular recordings were obtained from deep dorsal horn wide-dynamic range lamina V/VI neurones with receptive fields on the glabrous skin of the hind toes using 127-µm-diameter 2 MΩ parylene-coated tungsten electrodes (A-M Systems, Sequim, WA). The search stimulus consisted of light tapping of the hind paw as the electrode was manually lowered. Neurones were characterised from depths relating to the deep dorsal horn laminae (HFS 746 ± 100 μm; HFS (+CS) 708 ± 66 μm),^42^ and once a single unit was isolated, neurones were classified as wide dynamic range on the basis of sensitivity to dynamic brushing, noxious mechanical (128 mN), and noxious heat stimulation (48°C) of the receptive field. Data were captured and analysed by a CED Micro1401 interface coupled to a computer with Spike2 v4 software (Cambridge Electronic Design, Cambridge, United Kingdom). The signal was amplified (×7500), bandpass filtered (low-/high-frequency cut-off 0.5/2 kHz), and digitised at rate of 20 kHz.

Diffuse noxious inhibitory controls were recruited by applying 40 kPa pressure via a neonatal cuff to the gastrocnemius muscle contralateral to the neuronal recording as previously described.^5^ HFS was performed in independent experimental groups with and without application of the conditioning stimulus; animals were randomised into experimental groups with a random number generator. Electrical stimulation was delivered transcutaneously via 2 needles inserted either into the receptive field (HFS_RF_) or adjacent to the receptive field (HFS_RFA_; centre of the plantar surface between the footpads, a single 2-ms pulse was delivered to confirm correct placement). Five trains of electrical stimulation (100 Hz, 2 ms pulse width, 1-second duration) were delivered 10 seconds apart at 2 times the C-fibre threshold (via a DS3 Isolated Current Stimulator; Digitimer Ltd). Before HFS, receptive field maps were produced in response to 16, 64, and 128 mN pinprick stimulators (MRC Systems GmbH). An area was considered part of the receptive field if a response of 5 action potentials during 1-second stimulation was obtained. A rest period of 20 seconds between applications was used to avoid sensitisation. Receptive field sizes are expressed as a percentage area of a standardised paw measured using ImageJ (NIH, Bethesda, MD). The receptive field was subsequently stimulated with dynamic brushing (#2 squirrel hair artist's brush) and 16, 64, and 128 mN pinprick. Stimuli were applied 50 to 60 seconds apart for a duration of 10 seconds, and the evoked response was quantified. Baseline data represent mean of the 3 trials performed 5 minutes apart. After HFS, receptive field maps and stimulus-evoked neuronal responses were determined 15 and 45 minutes poststimulation (Fig. 1B).

### 2.5. Statistics

Statistical analyses were performed using SPSS v28 (IBM, Armonk, NY). The experimental unit for electrophysiological recordings was the individual rat; no animals were excluded from analysis. For psychophysical testing, the experimental unit was the individual subject; 45 were recruited but 8 were excluded from analysis because of withdrawal after the first session (3), incorrect performance with cuff algometry (3), equipment failure (1), and hyposensitivity after HFS (1). All data collection and analyses were performed unblinded. Mechanical coding of neurones was compared with a 2-way repeated-measures (RM) ANOVA, followed by a Bonferroni post hoc test for paired comparisons. Dynamic brush-evoked responses were compared with a 1-way RM ANOVA, followed by a Bonferroni post hoc test for paired comparisons. Where appropriate, sphericity was tested using Mauchly test; the Greenhouse–Geisser correction was applied if violated. Receptive field sizes were compared with a Friedman test followed by Wilcoxon post hoc with Bonferroni correction for paired comparisons. For psychophysical measures, data were compared with either a 1-way RM ANOVA or 2-way RM ANOVA followed by a Bonferroni post hoc test for paired comparisons. Multivariate linear regression was performed for correlation analysis followed by an ANOVA. Minimum group sizes were determined by a priori calculations using the following assumptions (α = 0.05, 1 − β = 0.8, ε = 1, effect size range *d* = 0.5-0.8). Effect sizes for mechanical hypersensitivity in electrophysiology experiments were guided by historical data, whereas effect sizes for human psychophysics were based on a pilot study (n = 16; data not included). Each rodent experimental group balanced the need to ensure statistical robustness while adhering to the “3 Rs” (refine, reduce, and replace; https://www.nc3rs.org.uk/the-3rs). All data represent mean ± 95% confidence interval (CI). Effect sizes (Cohen *d*) are reported in Table S1 (available as supplemental digital content at http://links.lww.com/PAIN/B983). *P < 0.05, **P < 0.01, ***P < 0.001.

## 3. Results

### 3.1. High-frequency electrocutaneous stimulation induces secondary but not primary hyperalgesia in humans

**Figure 2.**
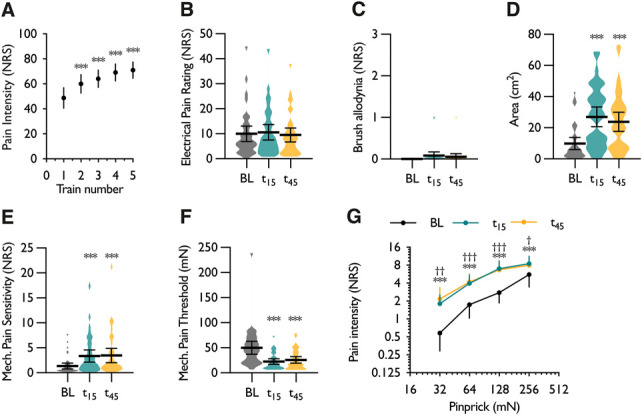
High-frequency electrocutaneous stimulation induces secondary pinprick hyperalgesia in humans. (A) Pain intensity ratings to repeated trains of electrical stimulation. (B) Pain intensity ratings to a single-pulse electrical stimulus (primary hyperalgesia). (C) Secondary brush allodynia. (D) Area surrounding the electrode in which stinging, pricking, or sharp sensations were experienced in response to a 10-g von Frey filament. (E) Secondary mechanical pain sensitivity. (F) Secondary mechanical pain threshold. (G) Pain intensity ratings to individual pinprick forces. Data represent mean ± 95% CI; n = 37. *P < 0.05, **P < 0.01, ***P < 0.001; For panels (A-E), “*” denotes difference from baseline. For panel (G), “*” denotes difference between baseline and 15 minutes time-point (t_15_), “†” denotes difference between baseline and 45 minutes time-point (t_45_). BL, baseline; NRS, numerical rating scale.

During high-frequency electrocutaneous stimulation in the control session, a temporal summation effect was observed with pain intensity ratings higher during the fifth stimulus train compared with the first (1-way RM ANOVA, *F*_1.38,49.73_ = 53.18, *P* = 5.23 × 10^−11^) (Fig. 2A). As a measure of primary hyperalgesia, electrical pain ratings (EPRs) were compared before and after HFS. Primary hyperalgesia was reported by 9 of 37 subjects, and overall, no time-dependent change in pain intensity ratings was observed (1-way RM ANOVA, *F*_1.55,55.62_ = 1.356, *P* = 0.267) (Fig. 2B). Secondary brush allodynia was infrequent and only reported by 3 of 37 subjects (1-way RM ANOVA, *F*_1.54,55.29_ = 2.423, *P* = 0.111) (Fig. 2C). Secondary pinprick hyperalgesia was more a prominent feature of sensitisation as evidenced by an increase in the area of secondary hyperalgesia in 28 of 37 subjects (1-way RM ANOVA, *F*_2,72_ = 21.787, *P* = 3.99 × 10^−8^) (Fig. 2D), an increase in mechanical pain sensitivity in 35 of 37 subjects (1-way RM ANOVA, *F*_1.60,57.42_ = 16.156, *P* = 0.00001) (Fig. 2E), and a decrease in mechanical pain threshold in 33 of 37 subjects (1-way RM ANOVA, *F*_1.18,42.42_ = 19.226, *P* = 0.00003) (Fig. 2F). Pain intensity ratings to increasing pinprick forces exhibited a linear stimulus–response relationship and were increased at both time-points post-HFS (2-way RM ANOVA, time × stimulus intensity interaction, *F*_3.43,123.4_ = 5.196, *P* = 0.00005) (Fig. 2F).

**Figure 3.**
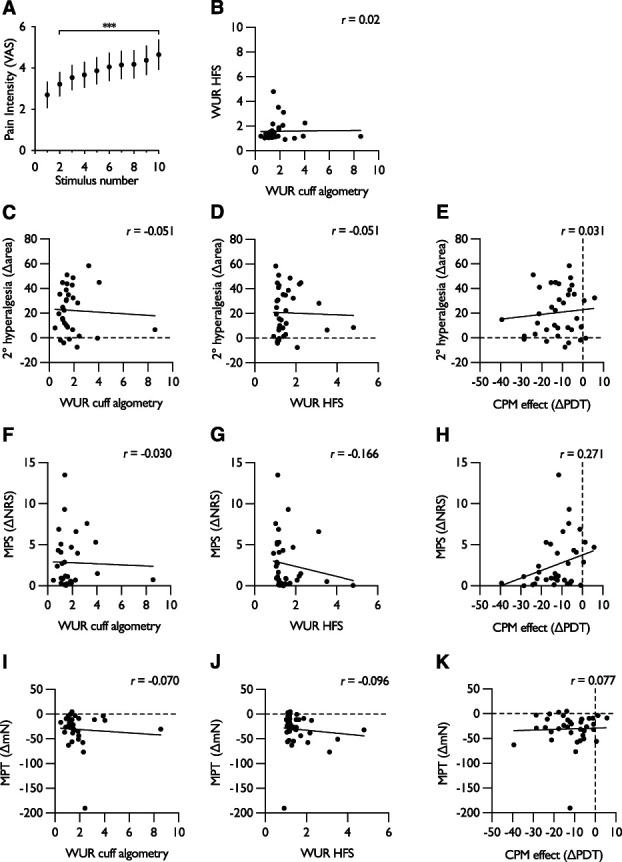
No correlation between wind-up ratios and CPM efficiency with measures of secondary hyperalgesia. (A) Temporal summation of pain in response to repetitive cuff pressure stimulation. (B) Correlation of electrical and cuff algometry wind-up ratios. (C) Correlation of cuff algometry wind-up ratio with area of secondary hyperalgesia. (D) Correlation of HFS wind-up ratio with area of secondary hyperalgesia. (E) Correlation of CPM effect with area of secondary hyperalgesia. (F) Correlation of cuff algometry wind-up ratio with mechanical pain sensitivity. (G) Correlation of HFS wind-up ratio with mechanical pain sensitivity. (H) Correlation of CPM effect with mechanical pain sensitivity. (I) Correlation of cuff algometry wind-up ratio with mechanical pain threshold. (J) Correlation of HFS wind-up ratio with mechanical pain threshold. (K) Correlation of CPM effect with mechanical pain threshold. For all dependent measures, peak change from baseline is used for comparison. For panel (A), data represent mean ± 95% CI; n = 32, ***P < 0.001; “*”denotes difference from first stimulus. 2°—, secondary, CPM, conditioned pain modulation; HFS, high-frequency stimulation; MPS, mechanical pain sensitivity; NRS, numerical rating scale; VAS, visual analogue scale; WUR, wind-up ratio.

Cuff pressure algometry was performed at baseline to assess temporal summation of pain, and a progressive increase in the pain intensity rating was observed from the 1st to the 10th stimulus (1-way RM ANOVA, *F*_2.31,83.01_ = 34.3, *P* = 1.48 × 10^−12^) (Fig. 3A). The wind-up ratio was calculated for both cuff algometry and electrical stimulation, and no correlation between the 2 was found (Fig. 3B). In addition, we examined whether wind-up ratios or CPM efficiency correlated with the degree of secondary hyperalgesia. The pressure/electrical wind-up ratios and the CPM effect did not correlate with a change in the area of secondary hyperalgesia (adj. *R*^2^ = −0.099; ANOVA *F*_3,28_ = 0.067, *P* = 0.977) (Figs. 3C–E), or with the change in mechanical pain sensitivity (adj. *R*^2^ = 0.037; ANOVA *F*_3,28_ = 1.402, *P* = 0.263) (Figs. 3F–H), or with the change in mechanical pain threshold (adj. *R*^2^ = −0.08; ANOVA *F*_3,28_ = 0.235, *P* = 0.872) (Figs. 3I–K). The development of secondary hyperalgesia was also not dependent on HFS intensity (mean 1.70 ± 0.35 mA) (Table S2, available as supplemental digital content at http://links.lww.com/PAIN/B983) or related to general health and state trait anxiety scores (Table S3, available as supplemental digital content at http://links.lww.com/PAIN/B983).

### 3.2. Applying a conditioning stimulus concurrent to high-frequency stimulation does not alter the development of secondary hyperalgesia in humans

**Figure 4.**
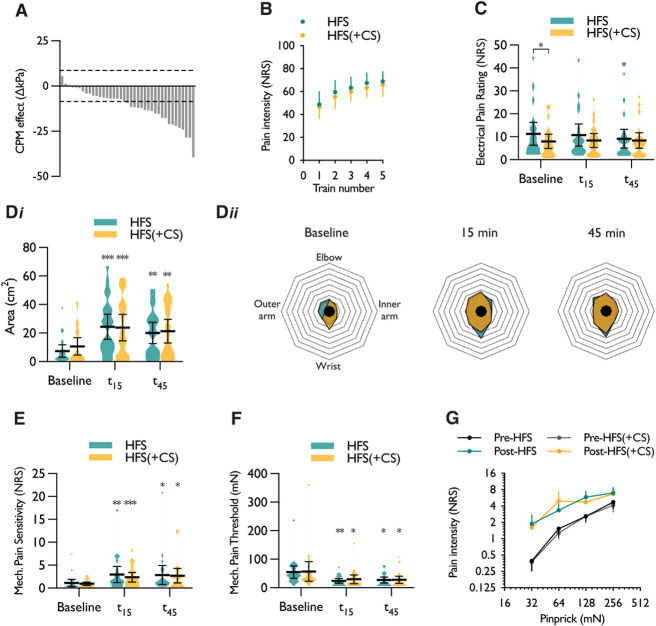
In CPM responders, applying a distant noxious conditioning stimulus during high-frequency electrocutaneous stimulation has no effect on the development of secondary hyperalgesia in humans. (A) Stratification of subjects based on CPM effect determined by a change in pain detection threshold. Bars represent individual responses, and dashed lines represent standard error of measurement (±8.64). (B) Pain intensity ratings to repeated trains of electrical stimulation with and without conditioning stimulus applied. (C) Pain intensity ratings to a single-pulse electrical stimulus (primary hyperalgesia). (D*i*) Area surrounding the electrode in which stinging, pricking, or sharp sensations were experienced in response to a 10-g von Frey filament, and (D*ii*) polygon plots of the mean spread of all subjects across experimental sessions. (E) Secondary mechanical pain sensitivity. (F) Secondary mechanical pain threshold. (G) Peak change of pain intensity rating to individual pinprick forces. Data represent mean ± 95% CI; n = 21. *P < 0.05, **P < 0.01, ***P < 0.001. Unless otherwise indicated, “*” denotes difference between time point and respective baseline. CPM, conditioned pain modulation; CS, conditioning stimulus; HFS, high-frequency stimulation; NRS, numerical rating scale.

Conditioned pain modulation was assessed with cuff pressure algometry to stratify the initial cohort (Fig. 4A). Based on a standard error of measurement of ±8.64, 21 of 37 subjects were classified as inhibitory CPM responders. Whole-group analyses are presented in Figure S2 (available as supplemental digital content at http://links.lww.com/PAIN/B983); however, in this subgroup of responders concurrently applying a distant noxious conditioning stimulus during high-frequency stimulation did not affect reported pain intensity ratings to the 5 electrical trains (2-way RM ANOVA, stimulus × CS interaction, *F*_2.65,53.07_ = 0.241, *P* = 0.845) (Fig. 4B). In the HFS (control) session, a time-dependent decrease in the primary pain intensity rating was observed at the 45 minutes time-point (1-way RM ANOVA, *F*_2,40_ = 4.74, *P* = 0.018); however, there was no change in the HFS (+CS) session (1-way RM ANOVA, *F*_1.28,25.75_ = 0.332, *P* = 0.625) (Fig. 4C). Comparing the interactive effect between experimental sessions revealed a difference in baseline electrical pain ratings (2-way RM ANOVA, time × CS interaction, *F*_2.,40_ = 4.393, *P* = 0.019) (Fig. 4C). A time-dependent increase in the area of secondary hyperalgesia was observed in both the HFS (control) (1-way RM ANOVA, *F*_1.57,31.42_ = 14.193, *P* = 0.0001) and HFS (+CS) sessions (1-way RM ANOVA, *F*_2,40_ = 10.97, *P* = 0.00016); however, applying a conditioning stimulus had no effect on the spread of secondary hyperalgesia (2-way RM ANOVA, time × CS interaction, *F*_2.,40_ = 0.527, *P* = 0.594) (Fig. 4D). A time-dependent increase in mechanical pain sensitivity was observed in both the HFS (control) (1-way RM ANOVA, *F*_1.49,29.87_ = 6.539, *P* = 0.0082) and the HFS (+CS) sessions (1-way RM ANOVA, *F*_1.4,28.00_ = 6.312, *P* = 0.011); however, the conditioning stimulus had no effect on mechanical pain sensitivity (2-way RM ANOVA, time × CS interaction, *F*_2.,40_ = 0.581, *P* = 0.564) (Fig. 4E). A time-dependent decrease in mechanical pain threshold was observed in both the HFS (control) (1-way RM ANOVA, *F*_1.13,22.57_ = 8.828, *P* = 0.0055) and the HFS (+CS) sessions (1-way RM ANOVA, *F*_1.06,21.22_ = 6.283, *P* = 0.019); however, the conditioning stimulus had no effect on mechanical pain thresholds (2-way RM ANOVA, time × CS interaction, *F*_1.15.,22.99_ = 0.09, *P* = 0.914) (Fig. 4F). When comparing the peak change in pain intensity ratings to individual pinprick forces, we found no evidence for an effect of the conditioning stimulus during HFS (3-way ANOVA, time × CS × stimulus intensity interaction, *F*_3,120_ = 1.112, *P* = 0.347) (Fig. 4G).

### 3.3. Rat spinal neurones exhibit distinct features of sensitisation after high-frequency stimulation when applied either directly to or adjacent to the receptive field

**Figure 5.**
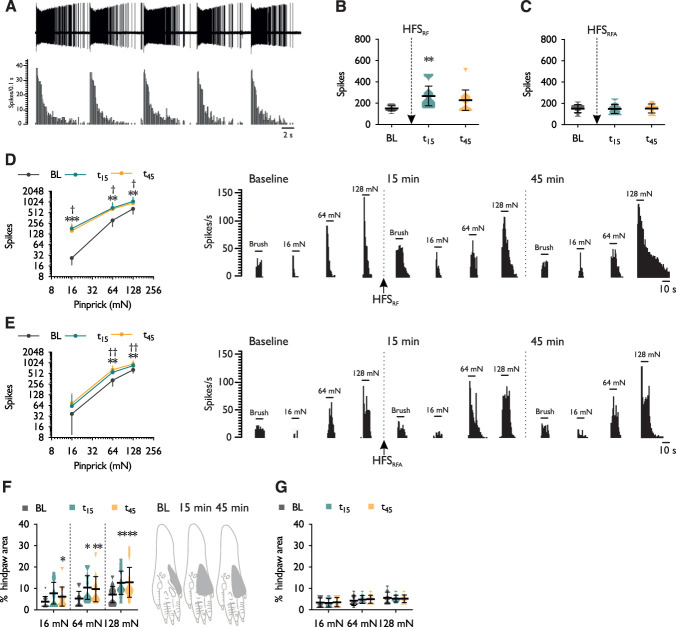
High-frequency electrocutaneous stimulation induces spinal neuronal sensitisation in rats. (A) Representative neurogram and histogram trace of a single-unit response to high-frequency electrocutaneous stimulation applied to the receptive field. Dynamic brush evoked neuronal responses before and after electrical stimulation applied either (B) directly to or (C) adjacent to the receptive field. Pinprick-evoked neuronal responses before and after electrical stimulation applied either (D) directly to or (E) adjacent to the receptive field. Receptive field sizes to pinprick stimuli before and after electrical stimulation applied either (F) directly to or (G) adjacent to the receptive field. Schematics of hind paws represent typical single-unit receptive field to 128 mN stimulation. Data represent mean ± 95% CI; *n*_HFS (RF)_ = 9, *n*_HFS (RFA)_ = 8. *P < 0.05, **P < 0.01, ***P < 0.001; “*” denotes difference between baseline and 15 minutes time-point (t_15_), “†” denotes difference between baseline and 45 minutes time-point (t_45_). HFS, high-frequency stimulation; RFA, receptive field adjacent; RF, receptive field.

Electrical stimulation was delivered transcutaneously in rats using identical stimulation parameters to the human study. When applied directly to the receptive field (HFS_RF_; Fig. 5A), the stimulation intensity (2 × C-fibre threshold; 1.76 ± 0.73 mA) was comparable with the human study (1.70 ± 0.35 mA). Electrical stimulation was also applied adjacent to the receptive field (HFS_RFA_) to study a neuronal correlate of secondary hyperalgesia. The stimulus intensity was fixed at 1.7 mA for all studied units based on the mean intensity obtained during quantitative sensory testing. HFS_RF_ produced a transient primary brush hypersensitivity (1-way RM ANOVA, *F*_2,16_ = 6.485, *P* = 0.0087) (Fig. 5B), which did not occur when HFS was delivered adjacent to the receptive field (1-way RM ANOVA, *F*_1.18,8.27_ = 0.066, *P* = 0.842) (Fig. 5C). Compared with brush hypersensitivity, HFS_RF_ and HFS_RFA_ produced a long-lasting primary (Fig. 5D) and secondary (Fig. 5E) pinprick hypersensitivity, respectively, to a range of non-noxious and noxious intensities of stimulation (2-way RM ANOVA, time × stimulus intensity interaction, HFS_RF_: *F*_4,32_ = 2.811, *P* = 0.042; HFS_RFA_: *F*_4,28_ = 4.278, *P* = 0.008). An expansion of receptive field size was observed after HFS_RF_ with increases in response to 16 mN (Friedman test, *P* = 0.006), 64 mN (Friedman test, *P* = 0.00086), and 128 mN (Friedman test, *P* = 0.00049) pinprick stimuli (Fig. 5F). In contrast, HFS_RFA_ had no impact on receptive field sizes to 16 mN (Friedman test, *P* = 0.264), 64 mN (Friedman test, *P* = 0.273) and 128 mN (Friedman test, *P* = 0.819) pinprick stimuli (Fig. 5G).

### 3.4. Applying a conditioning stimulus does not supress the development of high-frequency stimulation-induced primary or secondary spinal neuronal sensitisation in rats

**Figure 6.**
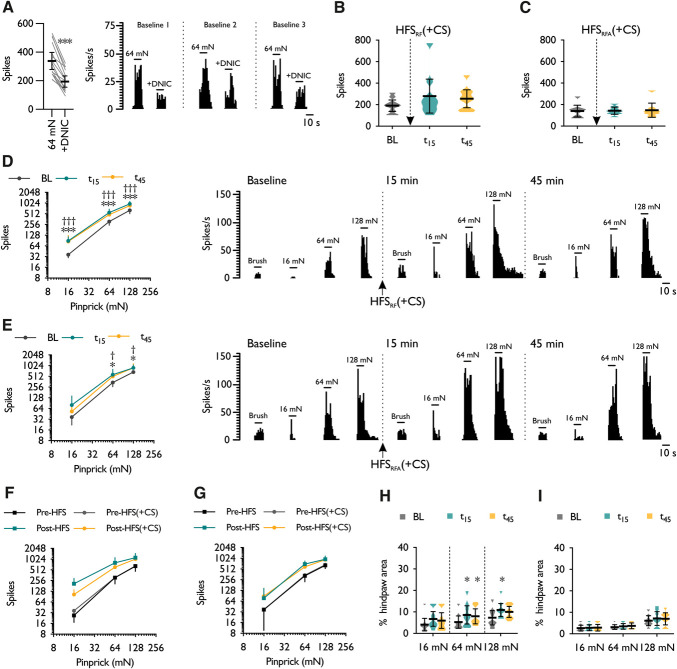
Applying a distant noxious conditioning stimulus during high-frequency electrocutaneous stimulation has no effect on the development of spinal neuronal sensitisation in rats. (A) Evoked neuronal responses to 64 mN stimulation with concurrent activation of DNIC (n = 17) and representative histogram trace of a single-unit response. Dynamic brush evoked neuronal responses before and after electrical stimulation applied either (B) directly to or (C) adjacent to the receptive field. Pinprick evoked neuronal responses before and after electrical stimulation applied either (D) directly to or (E) adjacent to the receptive field. (F) Peak change of primary pinprick hypersensitivity during HFS_RF_ and HFS_RF_ (+CS) experiments. (G) Peak change of secondary pinprick hypersensitivity during HFS_RFA_ and HFS_RFA_ (+CS) experiments. Receptive field sizes to pinprick stimuli before and after electrical stimulation applied either (H) directly to or (I) adjacent to the receptive field. Data represent mean ± 95% CI; *n*_HFS (RF)_ = 9, *n*_HFS (RFA)_ = 8. *P < 0.05, **P < 0.01, ***P < 0.001; “*” denotes difference between baseline and 15 minutes time-point (t_15_), and “†” denotes difference between baseline and 45 minutes time-point (t_45_). CS, conditioning stimulus; DNIC, diffuse noxious inhibitory controls; HFS, high-frequency stimulation; RFA, receptive field adjacent; RF, receptive field.

The development of primary and secondary neuronal sensitisation was assessed after the concurrent application of a distant noxious conditioning stimulus during delivery of HFS. For the HFS_RF_ study, the stimulation intensity did not differ from the control group (HFS_RF_: 1.76 ± 0.73 mA, HFS_RF_ (+CS): 1.18 ± 0.28 mA; unpaired *t* test with Welch correction, *P* = 0.176). The expression of DNIC was confirmed before all HFS (+CS) experiments; tonic pressure applied to the contralateral leg decreased neuronal firing (42.5 ± 5.86%) in response to 64 mN stimulation for all cells tested (paired *t* test, *P* = 8.27 × 10^−8^) (Fig. 6A). Primary brush hypersensitivity was less pronounced after HFS_RF_ when performed concurrently with a conditioning stimulus (1-way RM ANOVA, *F*_1.093,8.745_ = 2.926, *P* = 0.121) (Fig. 6B), and as before, brush-evoked responses remained comparable with baseline after HFS_RFA_ (+CS) (1-way RM ANOVA, *F*_2,14_ = 0.137, *P* = 0.874) (Fig. 6C). Primary (Fig. 6D) and secondary (Fig. 6E) pinprick hypersensitivity were still robustly induced after HFS (+CS) (2-way RM ANOVA, time × stimulus intensity interaction, HFS_RF_ (+CS): *F*_4,32_ = 3.805, *P* = 0.012; HFS_RFA_ (+CS): *F*_4,28_ = 2.79, *P* = 0.046). When comparing the time point of peak change from baseline, no differences in pinprick hypersensitivity were observed between the control and HFS (+CS) experiments when applied either directly to (Fig. 6F) or adjacent to (Fig. 6G) the receptive field (3-way ANOVA, HFS × CS × stimulus intensity interaction, HFS_RF_ (+CS): *F*_2,32_ = 0.390, *P* = 0.684; HFS_RFA_ (+CS): *F*_2,28_ = 0.789, *P* = 0.464). After HFS_RF_ (+CS), the expansion of receptive field size in response to 16 mN stimulation was less pronounced, and weak evidence was found for increased neuronal responsivity (Friedman test, *P* = 0.044; paired comparisons *P* > 0.05); however, increased responsivity to 64 mN (Friedman test, *P* = 0.004) and 128 mN (Friedman test, *P* = 0.0037) stimuli was still observed (Fig. 6H). The magnitude of change compared with the control experiment was similar (mean fold increase in RF size at 15 minutes: HFS_RF_ and HFS_RF_ (+CS), respectively, – 2.04 and 1.88 (16 mN), 2.62 and 1.66 (64 mN), 2.15 and 1.93 (128 mN)). As observed in the control experiment, receptive field sizes to 16 mN (Friedman test, *P* = 0.607), 64 mN (Friedman test, *P* = 0.097), and 128 mN (Friedman test, *P* = 0.565) pinprick stimuli were comparable with baseline after HFS_RFA_ (+CS) (Fig. 6I).

## 4. Discussion

In this translational study, wherein parallel human and rat protocols were applied, we investigated the interaction between neurophysiological responses in rats and perceptual responses in humans after HFS, and the development of secondary hyperalgesia or neuronal sensitisation and the activation of a descending inhibitory pathway when a second noxious conditioning stimulus was applied during HFS. High-frequency electrical stimulation enhanced perceptual responses to pinprick stimuli in cutaneous areas adjacent to the area of electrical conditioning. Subsequently, we observed that applying a conditioning stimulus concurrent to HFS stimulation did not affect the development of secondary hyperalgesia. The major advantage of our experimental design was that we were able to define the input–output relationship between peripheral stimuli and psychophysical responses in humans and spinal output in rats upon application of identical experimental paradigms. Human psychophysics and rat spinal neurones displayed similar stimulus–response relationships to pinprick stimulation before and after electrical conditioning. This alignment supports the translational value of the preclinical model to assess neural substrates of hyperalgesia, where second-order WDR neurones in the deep dorsal horn encode multiple features of nociceptive processing including fine-tuned intensity coding,^23^ spatial and temporal summation.^4,25^

As with previous reports,^3,17,46^ after HFS, we observed secondary hyperalgesia elicited by pinprick stimuli, but dynamic mechanical allodynia was infrequent. Secondary hyperalgesia was assessed using 3 measures (area, mechanical pain sensitivity, and mechanical pain threshold), and all subjects exhibited a change in at least one measure. We observed a similar time course of sensitisation, which follows early-LTP–like plasticity within spinal circuits when identical electrical stimulation is performed.^17,29^ Observations in rodents and corroborated by human studies confirm that C nociceptors within the primary area of electrical conditioning are “facilitators,” but A fibres adjacent to the area of electrical conditioning are “facilitated” leading to secondary hyperalgesia.^3,10^ Neuronal correlates of neurogenic hyperalgesia have been described within the spinothalamic tract revealing neuronal hyperexcitability within the primary area of inflammation and adjacent to inflammation.^27,36^ Likewise, we observe hyperexcitability of deep dorsal horn neurones to mechanical stimulation within the area of electrical conditioning and adjacent to the conditioning pathway, and these features of neuronal hyperexcitability were distinct. Brush hypersensitivity and an expansion of receptive field size were only observed when HFS was applied directly to the receptive field. An expansion of receptive field size indirectly indicates an increase in synaptic strength where previously subthreshold inputs on the firing fringe are now capable of evoking a suprathreshold response either through peripheral sensitisation or increased responsivity of dorsal horn neurones.^44^ Heterosynaptic facilitation of Aδ inputs may partially underlie hyperalgesia in regions immediately adjacent to the area of electrical conditioning.^40^ However, when HFS is delivered adjacent to the receptive field, an expansion of the receptive field is no longer apparent. At distant sites, descending mechanisms could predominate as facilitation of dorsal horn neuronal excitability to mechanical stimulation within adjacent areas can be attenuated by spinal transection or local anaesthetic block of the rostral ventromedial medulla.^27^ Although secondary neuronal hyperexcitability to thermal stimulation was absent in this latter study,^27^ Aδ- and C-fibre inputs are subjected to differential descending modulation,^24^ and secondary hyperalgesia may be driven by selective facilitation of Aδ-fibre inputs rather than by modality per se*.*^7,13^

We examined several factors that might account for some of the variation in the magnitude of secondary hyperalgesia observed. As we studied a healthy pain-free cohort, general health scores were high and anxiety scores were low, and perhaps unsurprisingly, these metrics did not correlate with measures of secondary hyperalgesia. We also assessed whether CPM efficiency or temporal summation of pain were contributing factors. Temporal summation of pain is interpreted as the capacity to amply nociceptive inputs, and spinal neuronal wind-up is considered the principal neuronal substrate. Wind-up represents a form of spinal plasticity that is distinct from central sensitisation but given that wind-up temporarily induces features of neuronal hyperexcitability reminiscent of central sensitisation, they are thought to share overlapping mechanisms.^11,21,43^ In a healthy cohort, we found no evidence that a low CPM response or a high temporal summation of pain ratio indicated higher susceptibility to secondary hyperalgesia induced by HFS. In individuals with pre-existing conditions such as osteoarthritis, high preoperative temporal summation of pain^28^ or low CPM efficiency^19^ identifies those at increased risk of developing higher postoperative pain. That said, in common with others,^15,16^ our study identified CPM nonresponders amongst the healthy population. Understanding whether, at the stage of first insult (as is the case with HFS application with or without conditioning), a reduced CPM efficiency lends itself to a preloading vulnerability to the development of chronic pain cannot be addressed by our study. However, because for CPM responders, HFS (+CS) did not influence the development of secondary hyperalgesia, the appropriate strategy for preventing pain development under these conditions seems to be dampening facilitation rather than enhancing inhibition. It is also noteworthy that if a pain state was already in place in the population we tested, the mechanistic underpinning of the physiological response would differ when hyperalgesia was induced; this distinction is important when considering pain relief targets.

During delivery of HFS, a temporal summation effect was also observed as the fifth stimulus train was rated more painful than the first. We found no evidence that the HFS temporal summation of pain ratio correlated with the degree of secondary hyperalgesia and, surprisingly, we found no correlation between the temporal summation of pain ratios evoked by cuff algometry and HFS. Both temporal summation and homotopic early LTP-like sensitisation have common mechanisms, eg, prevented by block of NMDA receptors,^18,30^ but this lack of correlation suggests that different amplification mechanisms are being recruited that may be a combination of peripheral, spinal, and descending factors. It is also feasible that the nature of the peripheral stimulus is a crucial determining factor as cuff algometry was performed at pain threshold levels of stimulation activating deep muscle inputs, whereas electrical stimulation was delivered transcutaneously at suprathreshold levels.

In our rat and human assessments, we confirmed that DNIC and CPM were effective at reducing spinal neuronal firing and increasing pain detection thresholds, respectively, when 2 acute noxious stimuli were applied. Our mean increase in pain threshold (11.7 kPa) was higher than previous studies applying an identical CPM paradigm.^6,12^ In addition, this method is comparable with cold pressor as an effective conditioning stimulus.^15^ The inhibitory effect of DNIC (42% decrease in firing) was also comparable with our previous studies where different test and conditioning stimuli were assessed.^2,5^ It is important to note that application of a conditioning stimulus in humans vs rats would evoke unique descending inhibitory mechanisms because DNIC are a neurophysiological correlate that assesses brainstem modulation with limited cortical involvement, whereas CPM is a perceptual endpoint encompassing “bottom-up” and “top-down” processing mechanisms and evaluates net inhibitory function extracted from gross descending output.^1^ One unresolved issue is whether CPM nonresponders reflect a failure to recruit this inhibitory pathway or whether inhibitory signalling is masked by descending facilitations. A caveat to interpreting the outcomes of this study is whether HFS (+CS) effectively induces CPM. In the CPM responders, when applied concurrently to HFS, a second distant conditioning stimulus did not affect the pain intensity ratings to the electrical stimulation or affect the development of hyperalgesia. This observation could relate to the relative intensity of the conditioning stimulus compared with the test stimulus as DNIC and CPM efficiency can vary with conditioning stimulus intensity.^9,20^ However, the frequency of the test stimulus is also a crucial factor as secondary hyperalgesia induced by low-frequency stimulation (10 Hz) was attenuated by application of a concurrent conditioning stimulus.^47^ Within bulbospinal circuits, at low stimulation frequencies, ie, the induction of wind-up, the recruitment of descending facilitatory pathways dominate^38^; however, with higher frequencies and intensities, ie, the induction of spinal LTP, net descending modulation is inhibitory.^8^ At these higher frequencies of stimulation, the activation of a unique inhibitory pathway (ie, DNIC) in addition to those phasically activated by the electrical stimulation seems to have limited capacity to summate and suppress the spread of sensitisation.

Successfully linking preclinical and clinical findings is essential for translation of pain research into improved treatment strategies, and differentiating between excitatory and inhibitory mechanisms is a key consideration when selecting the most appropriate drug target. This would be influenced by the stage (initiation, development, maintenance) of the physiological response to a noxious insult. Using electrophysiological recordings of spinal activity in rats and quantitative sensory testing in humans, we found that HFS induced features of secondary sensitisation in a manner that tracks across species. Our finding that excitatory signalling exceeds inhibitory controls during application of these experimental paradigms supports targeting of excitatory signalling to prevent the development of sensitisation. Inverting the traditional translation process in this way has the potential to provide insights into pain mechanisms, validation of therapeutic approaches, and optimisation of treatment algorithms.

## Conflict of interest statement

A.H.D. has received speaker and consultancy fees from Grünenthal and Oxford Cannabinoid Technologies.

## Appendix A. Supplemental digital content

Supplemental digital content associated with this article can be found online at http://links.lww.com/PAIN/B983.

## Supplementary Material

SUPPLEMENTARY MATERIAL
