# Supplementary material for: A back-translational study of descending interactions with the induction of hyperalgesia by high-frequency electrical stimulation in rats and humans
Source: Pain. 2024 Jan 9;165(9):1978–89. doi: 10.1097/j.pain.0000000000003166 (PMC11331830; doi:10.1097/j.pain.0000000000003166)
Supplement: SUPPLEMENTARY MATERIAL [file jop-165-1978-s002.pdf]

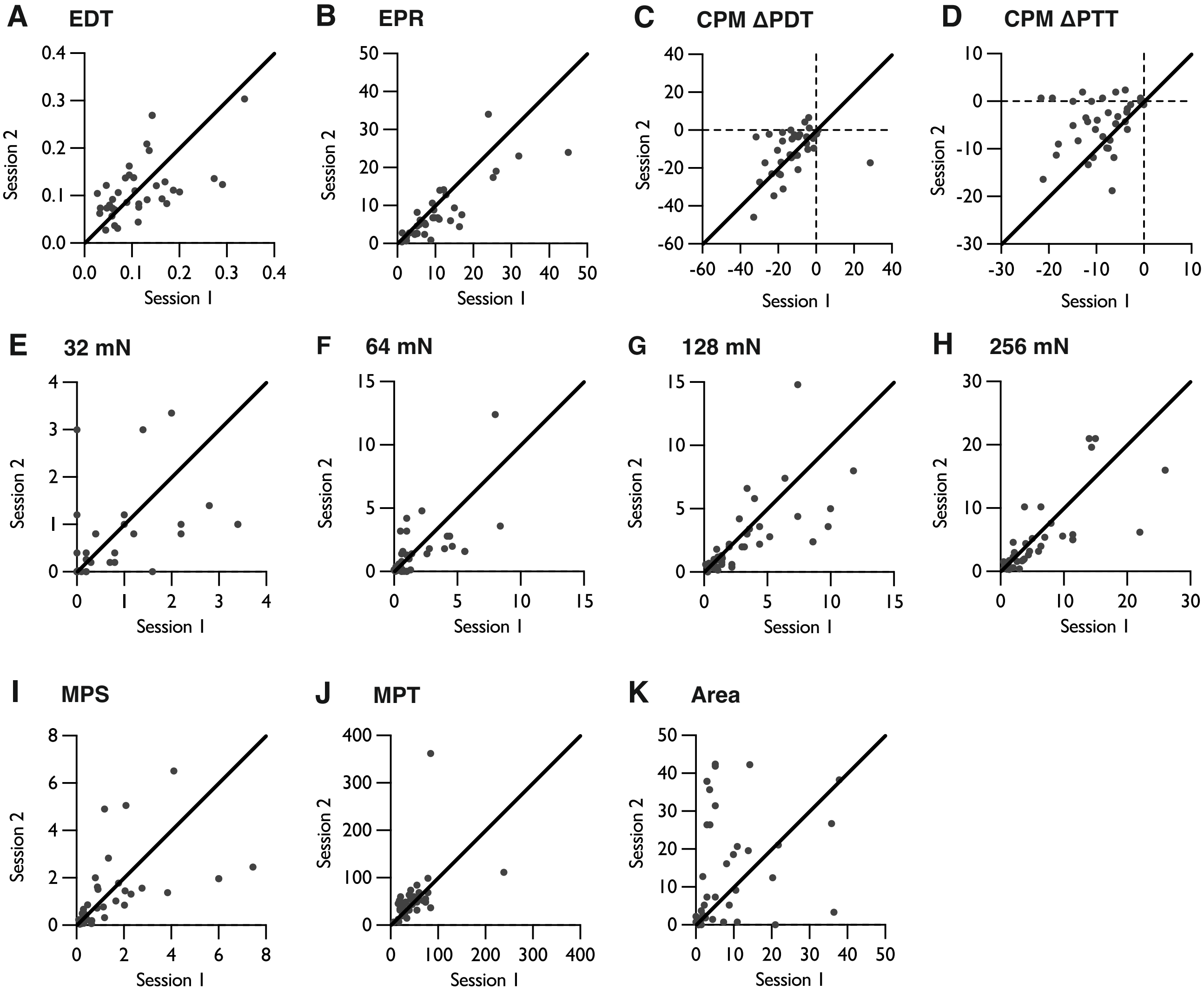

Supplementary Figure S1. Correlation of baseline measures in session 1 and session 2. (A) Electrical detection threshold (EDT) *r*=0.548**. **(B)** Electrical pain rating (EPR) to a single electrical pulse *r*=0.835**. **(C)** Conditioned pain modulation (CPM) effect on pain detection threshold (PDT) *r*=-0.423**. **(D)** CPM effect on pain tolerance threshold (PTT) *r*=-0.272. **(E)** Pain intensity rating to 32 mN pinprick *r*=0.835**. **(F)** Pain intensity rating to 64 mN pinprick *r*=0.680*. **(G)** Pain intensity rating to 128 mN pinprick *r*=0.685**. **(H)** Pain intensity rating to 256 mN pinprick *r*=0.745**. **(I)** Mechanical pain sensitivity (MPS) *r*=0.509**. **(J)** Mechanical pain threshold (MPT) *r*=0.414**. **(K)** Area of sensitivity to 10 g von Frey stimulation *r*=0.210. The black line indicates *y*=*x* and represents perfect equivalence between the sessions; *n*=37, **P*<0.05, ***P*<0.01.

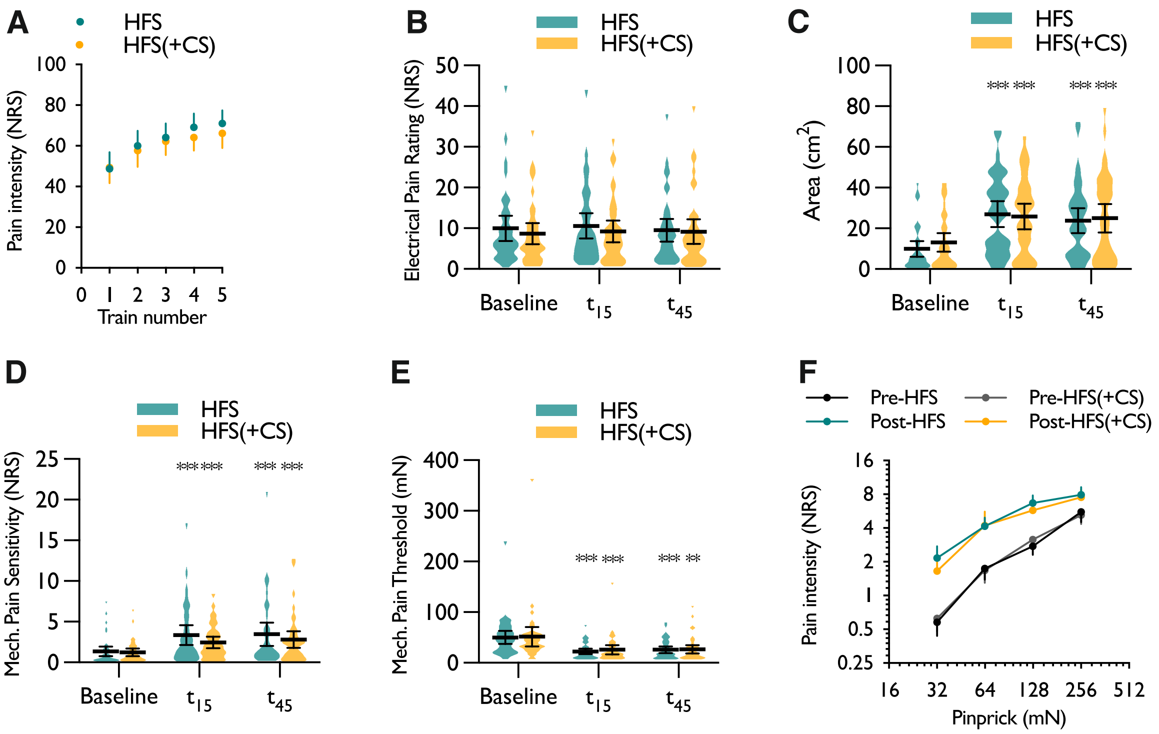

Supplementary Figure S2. Applying a distant noxious conditioning stimulus during high frequency electrocutaneous stimulation has no effect on the development of secondary hyperalgesia in humans. (A) Pain intensity ratings to repeated trains of electrical stimulation with and without conditioning stimulus applied. **(B)** Pain intensity ratings to a single pulse electrical stimulus (primary hyperalgesia). **(C)** Area surrounding the electrode in which stinging, pricking or sharp sensations were experienced in response to a 10 g von Frey filament. **(D)** Secondary mechanical pain sensitivity. **(E)** Secondary mechanical pain threshold. **(F)** Peak change of pain intensity rating to individual pinprick forces. Data represent mean ± 95% CI; n=37. **P<0.01, ***P<0.001; * denotes difference between time-point and respective baseline. CS – conditioning stimulus, HFS – high frequency stimulation, NRS – numerical rating scale.

Fig. 2B	t_{15}	t_{45}
EPR	0.063	-0.055

Fig. 2C	t_{15}	t_{45}
Brush allodynia	0.414	0.333

Fig. 2D	t_{15}	t_{45}
Area	1.078	0.901

Fig. 2D	t_{15}	t_{45}
MPS	0.701	0.647

Fig. 2D	t_{15}	t_{45}
MPT	-0.922	-0.783

Fig. 2E	t_{15}	t_{45}
32 mN	0.731	0.611
64 mN	0.646	0.658
128 mN	0.773	0.738
256 mN	0.386	0.330

Fig. 4B	
1	-0.092
2	-0.177
3	-0.171
4	-0.210
5	-0.147

Fig. 4C	t_{15}	t_{45}
HFS	-0.050	-0.215
HFS(+CS)	-0.058	-0.052

Fig. 4D	t_{15}	t_{45}
HFS	1.121	0.952
HFS(+CS)	0.762	0.668

Fig. 4E	t_{15}	t_{45}
HFS	0.614	0.502
HFS(+CS)	0.830	0.694

Fig. 4F	t_{15}	t_{45}
HFS	-0.847	-0.737
HFS(+CS)	-0.318	-0.280

Fig. 4G	HFS	HFS(+CS)
32 mN	0.504	0.728
64 mN	0.505	0.467
128 mN	0.591	0.540
256 mN	0.327	0.399

Fig. 5B	t_{15}	t_{45}
HFS(RF)	1.337	0.858

Fig. 5C	t_{15}	t_{45}
HFS(RFA)	-0.048	0.025

Fig. 5D	t_{15}	t_{45}
16 mN	3.818	1.546
64 mN	1.454	1.212
128 mN	1.121	1.042

Fig. 5E	t_{15}	t_{45}
16 mN	0.406	0.507
64 mN	1.583	1.740
128 mN	1.207	1.345

Fig. 5F	t_{15}	t_{45}
16 mN	0.758	0.496
64 mN	0.866	0.706
128 mN	0.893	0.778

Fig. 5G	t_{15}	t_{45}
16 mN	-0.042	0.075
64 mN	0.244	0.289
128 mN	-0.167	-0.189

Fig. 6A	
DNIC	-1.515

Fig. 6B	t_{15}	t_{45}
HFS_{RF} +CS	0.585	0.709

Fig. 6C	t_{15}	t_{45}
HFS_{RFA} +CS	0.070	0.119

Fig. 6D	t_{15}	t_{45}
16 mN	2.033	1.247
64 mN	1.695	1.759
128 mN	1.804	1.090

Fig. 6E	t_{15}	t_{45}
16 mN	0.773	0.683
64 mN	1.090	1.042
128 mN	1.221	0.869

Fig. 6F	HFS_{RF}	HFS_{RF}(+CS)
16 mN	2.363	2.035
64 mN	1.616	2.745
128 mN	1.311	2.209

Fig. 6G	HFS_{RFA}	HFS_{RFA}(+CS)
16 mN	0.649	0.970
64 mN	2.331	1.226
128 mN	1.488	1.172

Fig. 6H	t_{15}	t_{45}
16 mN	0.634	0.442
64 mN	0.703	0.641
128 mN	0.867	0.711

Fig. 6I	t_{15}	t_{45}
16 mN	0.091	0.101
64 mN	0.227	0.395
128 mN	0.313	0.285

Supplementary Table S1. Effect sizes (Cohen's *d*) denote change from respective baseline or control. CS – conditioning stimulus, EPR – electrical pain rating, HFS – high frequency stimulation, MPS – mechanical pain sensitivity, MPT – mechanical pain threshold, RF – receptive field, RFA – receptive field adjacent.

r =	HFS Intensity (mA)	Mean HFS(control) NRS
**Area 15 min**	0.240	0.294
**Area 45 min**	0.386*	0.302
**MPS 15 min**	-0.0941	0.524**
**MPS 45 min**	-0.0244	0.461**
**MPT 15 min**	-0.218	-0.116
**MPT 45 min**	-0.191	-0.0886

Supplementary Table S2. Correlations between the intensity of the HFS trains (mA) and the mean pain intensity ratings for five electrical HFS trains (NRS) with each of the dependant measures at each timepoint (calculated as difference from baseline) in the HFS(control) session; n=37, *P<0.05, **P<0.01. HFS – high frequency stimulation, MPS – mechanical pain sensitivity, MPT – mechanical pain threshold, NRS – numerical rating scale.

r =	Physical Functioning	Role limitations due to physical health	Role limitations due to emotional problems	Energy/ fatigue	Emotional well-being	Social functioning	Pain	General Health	State	Trait
**Area 15 min**	0.173	0.238	-0.077	-0.079	-0.108	-0.120	0.287	-0.053	0.402	0.153
**Area 45 min**	0.302*	0.209	-0.013	-0.004	-0.073	-0.010	0.328*	-0.015	0.386**	0.162
**MPS 15 min**	0.142	0.088	0.136	-0.032	-0.066	0.042	-0.024	0.133	0.098	0.089
**MPS 45 min**	0.162	0.110	0.244	0.067	0.054	0.156	0.154	0.213	-0.013	-0.002
**MPT 15 min**	0.098	0.032	-0.206	-0.235	-0.230	-0.276	-0.179	-0.313	0.327	0.366
**MPT 45 min**	-0.092	0.057	-0.168	-0.136	-0.027	-0.134	0.028	-0.089	0.112	0.071

Supplementary Table S3. Correlations between scores for general health and state trait anxiety with each of the dependant measures at each timepoint (calculated as difference from baseline) in the HFS(control) session; *n*=37, **P*<0.05, ***P*<0.01. HFS – high frequency stimulation, MPS – mechanical pain sensitivity, MPT – mechanical pain threshold.
